# Scoring System Based on RNA Modification Writer-Related Genes to Predict Overall Survival and Therapeutic Response in Bladder Cancer

**DOI:** 10.3389/fimmu.2021.724541

**Published:** 2021-08-26

**Authors:** Pu Zhang, Zijian Liu, Decai Wang, Yunxue Li, Yifei Xing, Yajun Xiao

**Affiliations:** ^1^Department of Urology Surgery, Union Hospital, Tongji Medical College, Huazhong University of Science and Technology, Wuhan, China; ^2^Department of Head and Neck Oncology and Department of Radiation Oncology, Cancer Center and State Key Laboratory of Biotherapy, West China Hospital, Sichuan University, Chengdu, China; ^3^Department of Emergency Surgery, Union Hospital, Tongji Medical College, Huazhong University of Science and Technology, Wuhan, China

**Keywords:** RNA modification “writer”, bladder cancer, tumor microenvironment, tumor mutation burden, immunotherapy

## Abstract

**Introduction:**

It’s widely reported the “writer” enzymes mediated RNA adenosine modifications which is known as a crucial mechanism of epigenetic regulation in development of tumor and the immunologic response in many kinds of cancers. However, the potential roles of these writer genes in the progression of bladder cancer (BLCA) remain unclear.

**Materials and Methods:**

We comprehensively described the alterations of 26 RNA modification writer genes in BLCA from the genetic and transcriptional fields and identified writer-related genes from four independent datasets. Utilizing least absolute shrinkage and selection operator (LASSO) regression and multivariate Cox regression, we constructed a ten writer-related gene signature. After that, we confirmed the predictive and prognostic value of this signature on another six independent datasets and established a nomogram to forecast the overall survival (OS) and mortality odds of BLCA patients clinically.

**Results:**

The writer-related genes signature showed good performance in predicting the OS for BLCA patients. Moreover, the writer-related gene signature was related to EMT-related pathways and immune characteristics. Furthermore, the immune cell infiltration levels of CD8 T cells, cytotoxic cells, M1/2 macrophage cells and tumor mutation burden might be able to predict which patients will benefit from immunotherapy. This could also be reflected by the writer-related gene signature.

**Conclusions:**

This signature might play an important role in precision individualized immunotherapy. The present work highlights the crucial clinical implications of RNA modifications and may help developing individualized therapeutic strategies for patients with BLCA.

## Introduction

With high morbidity and mortality rates, Bladder cancer (BLCA) is one of the most malignant and highly aggressive tumors in the urinary system (1). Owing to the 2020 China cancer statistics, it was reported that 81,400 cases of BLCA were diagnosed and that BLCA was the cause of death for 17,980 cases in the US in 2020 (2). Based on the histological differentiation of BLCA, the tissue can be divided into low grade with a good prognosis and high grade with a poor prognosis. Based on whether the tissue invades the muscle of the bladder, BLCA can be generally divided into a non-muscle-invasive or muscle-invasive disease that is prone to relapse and metastasis (3). In the past few decades, although there have been many well-established surgical and chemotherapy options for different subtypes of BLCA, the recurrence and mortality rates of BLCA have remained high. The specific genetic or epigenetic regulatory mechanisms during the progression and development of BLCA still need further investigation to form a solid theoretical basis for eradicating this kind of tumor in the future.

Previous studies have suggested that genetic mutations in some chromosomal genes, such as FGFR3, RB1, HRAS, TP53, and TSC1, lead to bladder tumors, and these genes play an important role in the regulation of cell division, which prevents cells from dividing too quickly (4). Somatic mutation might be one of the important components in the development of BLCA, while not altering the nucleotide sequence of genes, epigenetics studies stable phenotypes led by changes of chromosome (5). RNA modification is commonly seen among all nucleotides. At the RNA level there are over 170 modifications, consisting of m5C, m3C, m7G, and Nm modifications (6, 7). We put stress on modifications of adenine-related RNA, because adenine is the nucleotide that is most frequently modified. The modifications are primarily led by the activity of enzymes called “writer” genes.

The sixth and first nitrogen atoms of the adenine base were influenced by the modification of m6A and m1A. And all of them could contribute to significant changes in some cellular processes, playing a major part in some unnormal conditions such as the occurrence of tumor (8, 9). Working as the RNA-processing mechanism, APA generates transcripts owning various lengths of 3′-untranslated regions (UTRs) or coding regions (10). Catalyzed by ADAR enzymes, A-to-I is a common kind of RNA editing that is a well-known posttranscriptional mechanism altering nucleotides in the transcripts (11). All of these RNA edits may finally cause the difference of the sequence of amino acids and impact transcriptional processes, thus leading to tumor formation or invasion through modifications of tumor-associated genes.

To comprehensively explore the importance of posttranscriptional modifications in progression and metastasis of BLCA, the investigation of these RNA alterations is urgently needed. It has been reported that m6A-related genes are differentially expressed in BLCA and could serve as reliable prognostic biomarkers (12). Only a few studies have reported on the function of m1A, APA and A-I RNA editing in BLCA, and the functions of RNA editing are indeed very important in research on other kinds of cancer. Machine learning models based on gene expression are widely used in the prognostic detection of diseases and drug efficacy. For BLCA, multiple studies have been investigated to establish prognostic signatures according to the expression levels of immune-associated genes (13, 14), EMT-related genes (15) or lncRNAs (16, 17). However, all of them failed to further investigate the underlying mechanism of the prognostic signatures, and no signatures were based on adenosine modification-related genes.

In this study, we summarized four main types of adenosine modifications, namely, m6A, m1A, APA and A-I RNA editing, and depicted the alteration landscape. Through the expression levels of these writer-related genes, we recognized RNA modification writer-related genes and established a prognostic signature. Furthermore, we investigated the underlying mechanism of the signature and found that poor prognosis was associated with activated EMT-related pathways prone to metastasis, while low risk was related to higher levels of cytotoxic cells and CD8 T cells infiltration, which might be the response to immunotherapy. Finally, we verified the efficacy of the signature using various external datasets and established a risk assessment nomogram.

## Materials and Methods

### Data Collection and Preprocessing

Public gene expression data and complete clinical information were retrieved from the Gene-Expression Omnibus (GEO) and The Cancer Genome Atlas (TCGA) databases. Totally, 7 eligible BLCA cohorts [GSE13507 (18), GSE32548 (19), GSE32894 (20), GSE48075 (21), GSE70691 (22), GSE31684 (23) and TCGA-BLCA (The Cancer Genome Atlas-Bladder Carcinoma)] were used in the research for bioinformatic analysis. The clinical baseline information of bladder cancer patients involved in this study was shown in **Table 1** and detailed clinical data of all patients were shown in **Table S1**. When it came to the microarray data deriving from the GEO database, we directly downloaded the normalized matrix files. For datasets in TCGA, we downloaded RNA sequencing data (FPKM values) of gene expression and copy number variation (CNV) data from the XENA database (https://xenabrowser.net/datapages/), and somatic mutation data were downloaded using the R package TCGAbiolinks (24). Then, we transformed FPKM values into transcripts per kilobase per million (TPM) values. The RNA modification writer genes included m1A modification genes, m6A modification genes, APA modification genes, and A-I RNA editing genes obtained from a previously published study (25).

**Table 1:** Clinical characteristics of bladder cancer patients.

	GSE13507	GSE32548	GSE32894	GSE48075	GSE70691	GSE31684	TCGA
**Age, Median (range)**	66 (24, 88)	70 (38, 90)	71 (20, 96)	68.8 (42.7, 89)	NA	69.17 (41.73, 91.08)	69 (34, 90)
**Gender**							
Male	135 (81.8%)	114 (78.1%)	228 (74.0%)	52 (75.4%)	NA	68 (73.1%)	308 (73.9%)
Female	30 (18.2%)	32 (21.9%)	80 (26.0%)	17 (24.6%)	NA	25 (26.9%)	109 (26.1%)
**Stage**							
I/II	129 (78.2%)	104 (71.2%)	280 (90.9%)	111 (78.2%)	NA	32 (34.4%)	134 (32.3%)
III/IV	36 (21.8%)	42 (28.8%)	28 (9.1%)	31 (21.8%)	NA	61 (65.6%)	281 (67.7%)
**Grade**							
Low	105 (63.6%)	65 (45.5%)	151 (49.3%)	NA	NA	6 (6.5%)	21 (5.1%)
High	60 (36.4%)	81 (55.5%)	155 (51.7%)	NA	NA	87 (93.5%)	393 (94.9%)

NA, not applicable.

### Construction of a Writer-Related Gene Signature of BLCA

Correlation coefficients between the RNA modification writer genes and potentially regulated mRNAs were computed by Spearman correlation analyses. The genes with correlation coefficients > 0.4 were deemed writer-related genes, and these genes from four different datasets were combined as candidate writer-related genes for deeper analysis. With the expression profiles of the candidate writer-related genes, we conducted least absolute shrinkage and selection operator (LASSO) regression analysis (26). It could help solve the problem of collinearity of a large number of gene expression values, to identify the most representative prognostic genes in the TCGA dataset. Based on the candidate genes generated *via* the above filtering process, a model was at last employed to build a prognostic signature. Utilizing the coefficients deriving from the multivariate Cox regression, we created the risk score formula through the equation: Risk Score = (Coef _i_ × Exp _i_). We used receiver operating characteristic (ROC) curves to judge the efficiency of the signature. All the analysis were completed in the R environment using specific R packages.

### Functional Annotation and Gene Set Variation Analysis

Gene ontology analysis and Kyoto Encyclopedia of Genes and Genomes (KEGG) pathway analysis were completed by utilizing DAVID (david.ncifcrf.gov) to identify the functions of candidate writer-related genes, and the online tool Image GP was utilized to show the outcomes of the GO and KEGG analyses (http://www.ehbio.com/ImageGP/). To investigate the underlying mechanism between the different risk groups, GSVA was performed to conduct (27) with hallmark gene sets deriving from the MSigDB database.

### Construction of a Nomogram According to the Gene Signature and Clinical Traits

We performed Univariate and multivariate Cox regression analyses to select independently prognostic indicators combined with clinical traits and risk scores calculated by gene signatures. Then using the “rms” R package, a nomogram encompassing the risk score and clinicopathological traits was constructed to forecast the survival possibility and mortality odds. Using a calibration plot, predictive accuracy was tested.

### Estimation of TME Cell Infiltration Abundance

The CIBERSORT was employed to calculate the infiltration levels of 22 types of immune cells in BLCA following the instructions from Newman et al. (28), with a 1000-permutation test and samples with p > 0.05 removed before further analysis. Furthermore, the levels of immune cell infiltration in the BLCA tumor microenvironment was also determined utilizing a single-sample gene set enrichment analysis (ssGSEA) algorithm, and the sets of immune cell markers independently published in articles were included in our study (**Table S2**) (29).

### Prediction of Immunotherapy Response in Patients

According to tumor pretreatment expression profiles, the Tumor Immune Dysfunction and Exclusion (TIDE) database can evaluate multiple published transcriptomic biomarkers to predict the immunotherapy response of patients (http://tide.dfci.harvard.edu/) (30). The input data should be normalized, and the recommended tumor types of this website were melanoma and non-small cell lung cancer (NSCLC); The analysis results of this website could only play an auxiliary role in our research. The TIDE value was calculated and used to assess the probability of immunotherapy response, and the cutoff of the TIDE value was set to a default of 0.

### Chemotherapeutic Response Prediction

The chemotherapeutic response for each sample was predicted according to the largest public pharmacogenomics database, the Genomics of Drug Sensitivity in Cancer (GDSC), (https://www.cancerrxgene.org/). Six usually adopted chemotherapy drugs in bladder cancer, namely, cisplatin, gemcitabine, doxorubicin, methotrexate, paclitaxel and vinblastine, were selected for use. We used R package to implement “pRRophetic the prediction process, where the samples’ half-maximal inhibitory concentration (IC50) was evaluated following the instructions described previously (31).

### Statistical Analysis

We conducted spearman correlation analysis to evaluate the correlation coefficient among each pair of indicators in this study. The Wilcoxon test was conducted to contrast the variation between pairs of distinctive groups. Based on the association between the risk score and the survival time of patients, we employed the “survminer” package to confirm the cutoff threshold with survival information. This was employed to dichotomize the risk score and expression of writer genes, and all latent cutoff points were repeatedly confirmed to identify the maximum rank statistic. Then according to the maximum selected log-rank statistics, the patients were divided into two groups. All statistical tests were two-sided. P < 0.05 was believed statistically significant.

## Results

### Genetic and Transcriptional Alteration Landscape of Four Types of RNA Modification Writer Genes in BLCA

**Figure 1:**
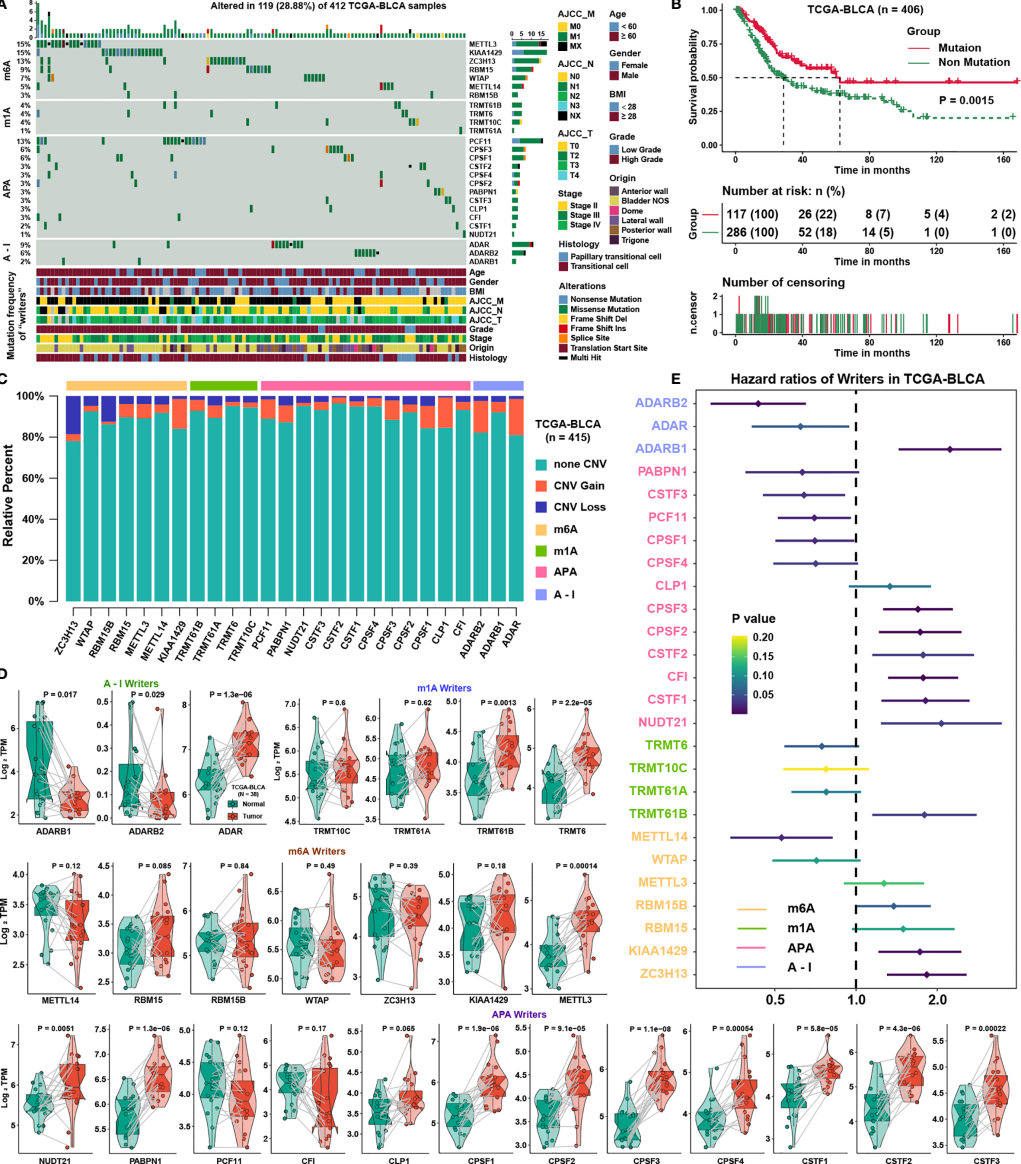
Genetic and transcriptional alterations of RNA modification writer genes in BLCA. **(A)** The mutation frequency of 26 RNA modification writers in 412 BLCA patients from the TCGA cohort. **(B)** Kaplan-Meier curves showing the overall survival of patients with or without mutations in RNA modification writer genes in the BLCA cohort. p < 0.05 in the two-sided log-rank test was considered statistically significant. **(C)** Bar graphs showing the frequency of CNV gain, loss and non_CNV of RNA modification writer genes in the TCGA-BLCA cohort. The height of each bar represents the alteration frequency. **(D)** Box plots show the expression distribution of 26 writer genes of 4 types of RNA modifications between paired normal and BLCA tissues. **(E)** The prognostic analyses for 26 writer genes in TCGA; a hazard ratio >1 represents a risk factor and a hazard ratio <1 represents a protective factor for survival.

We evaluated the levels of nonsilent somatic mutations in 26 writers to determine genetic alterations in RNA modification writer genes in BLCA. Of all the 412 BLCA samples in TCGA, 119 (28.88%) experienced mutations of RNA modification writer genes (**Figure 1A**). METTL3 and KIAA1429 had the highest mutation frequency (15%), followed by ZC3H13 and PCF11. However, BLCA patients without mutations of these writers experienced worse overall survival than those with such mutations (**Figure 1B**; log-rank test, p = 0.0015), suggesting genetic alterations of writer genes may have a useful role in BLCA. Then, we explored somatic copy number alterations of those writer genes and discovered ADAR, ADARB2, CLP1 and KIAA1429 had widespread copy number variation (CNV) gains, while ZC3H13 and RBM15B had the highest frequency of CNV loss (**Figure 1C**). We compared the gene expression levels between paired normal and tumor samples to confirm if those genetic variations influenced the expression levels of writer genes in patients with BLCA and demonstrated that the expression of many of the writer genes was significantly elevated in BLCA (**Figure 1D**). Writers with CNV gain, such as ADAR and CPSF1/3, were highly expressed in BLCA samples than in normal bladder samples, suggesting that CNV may be a regulatory factor of these writer genes. However, some writers with a high frequency of CNV gain or loss showed no change between normal and tumor samples, meaning that CNV was not the only factor determining the expression of writers. Based on the “surv-cutpoint” function, the hazard ratios of writers were calculated in the case of the best separated groups in BLCA (**Figure 1E**). Some writers with higher expression in BLCA were also risk factors, such as TRMT61B, NUDT21, CSTF1/2, and CPSF2/3, while ADARB2 was the only protective factor with lower expression in BLCA. The analysis showed great difference of the both the genetic landscape and expression levels of RNA modification writer genes between normal and BLCA patients, showing RNA modification writer genes has latent function in the oncogenesis of BLCA.

### Identifying Representative Candidate Prognostic Writer-Related Genes

**Figure 2:**
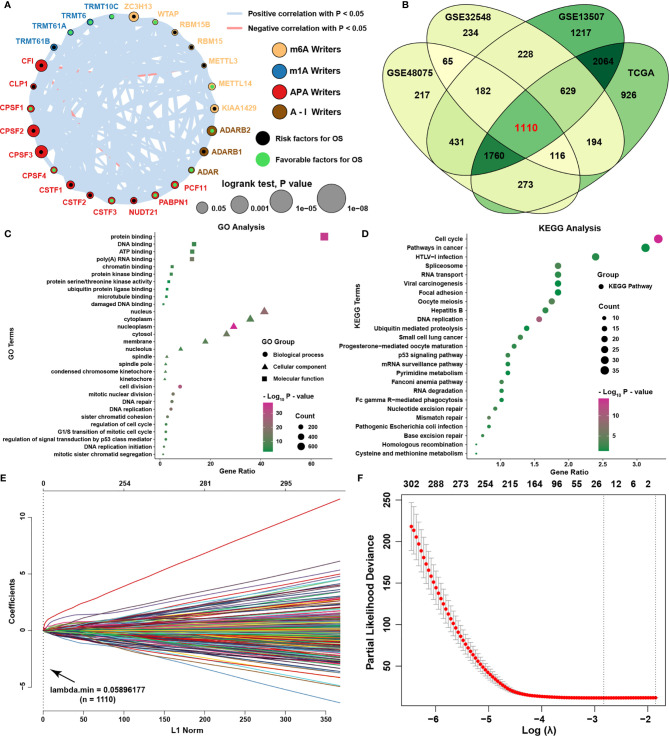
Identifying representative candidate prognostic writer-related genes. **(A)** The interactions among writer genes in BLCA. The lines linking regulators show their interactions, and line thickness shows the correlation strength between regulators. A negative correlation is marked with red, and a positive correlation is marked with gray. **(B)** Venn diagram for writer-related genes in four independent datasets. **(C, D)** GO and KEGG analyses of writer-related genes and terms are shown on the left. **(E, F)** Ten‐fold cross‐validation for tuning parameter selection in the LASSO model and the LASSO coefficient profiles of candidate genes are shown.

The comprehensive landscape of writer gene interactions and their prognostic value for BLCA in TCGA was demonstrated by a writer gene network (**Figure 2A**, **Table S3**). We discovered that the expression levels of writer genes were prone to be positively related to each other, meaning that the four types of RNA modification writer genes may have a significant role in the regulation and modification of RNA. To identify the candidate genes regulated by writer genes for further functional prediction of writer genes, correlation analysis was conducted between the writer genes and other protein-coding genes. We identified 1110 genes correlated with the expression of writer genes with an |correlation coefficient| > 0.4 in four independent datasets (**Figure 2B**, **Tables S4–8**). GO analysis showed that potentially modified genes were highly associated with protein and DNA binding, cell division, and DNA replication-related biological functions (**Figure 2C**). KEGG analysis showed that cell cycle, pathways in cancer and viral carcinogenesis were highly enriched (**Figure 2D**). These results indicated that writer genes might influence protein or DNA binding to regulate the cell cycle, division and cancer-related viral infection or pathways in the progression and metastasis of BLCA. For further selection of the most prognostic valuable candidate writer-related genes, a set of 15 candidate writer-related genes were identified with the LASSO algorithm to for further analysis (**Figures 2E, F**).

### Constructing a Writer-Related Gene Signature of BLCA

**Figure 3:**
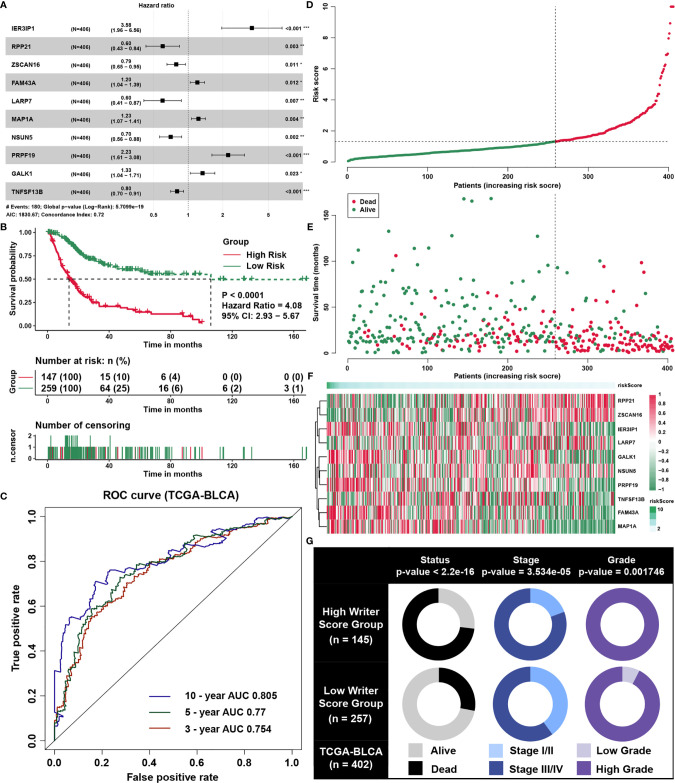
Construction of a writer-related gene signature of BLCA. **(A)** Hazard ratio and P‐value of constituents involved in multivariate Cox regression and some parameters of the gene signature. **(B)** Kaplan‐Meier survival curves plotted to estimate the overall survival probabilities for the low‐risk *versus* high‐risk group. **(C)** ROC curves plotted for 3‐, 5‐ and 10‐y overall survival. **(D)** The gene signature risk score distribution. **(E)** The vital status of patients in the high‐risk and low‐risk groups. **(F)** Heatmap of the expression profiles of members in the gene signature. **(G)** The correlations between risk score and clinical parameters. Chi-square tests were used to test for statistical correlations. *P < 0.05, **P < 0.01, ***P < 0.001.

Eventually, we found ten writer-related genes with multivariate Cox regression analysis to build a predictive signature in the TCGA dataset (**Figure 3A**, **Table S9**). The signature’s concordance index was 0.72, and the P‐value = 5.7099e−19. Using the risk score formula from multivariate Cox regression, we calculated the risk score of each patient. We divided all patients to two groups (High *VS* Low) according to the best cutoff point of the risk scores. The patients having lower risk scores owned better survival time (**Figure 3B**). The AUC of the signature for survival of three years, five years and ten years was 0.754, 0.77 and 0.805 respectively (**Figure 3C**). Moreover, the number of surviving patients fell and cancer‐associated death enhanced with rising risk score. We showed every candidate gene expression value in the formula related to the risk score using the heatmap (**Figures 3D–F**). We also discovered that patients with stage III/IV, which were considered risk factors in the clinical setting, were more likely to be involved in the group of high risk than in the group of low risk (**Figure 3G**).

### Building a Predictive Nomogram and External Validation

**Figure 4:**
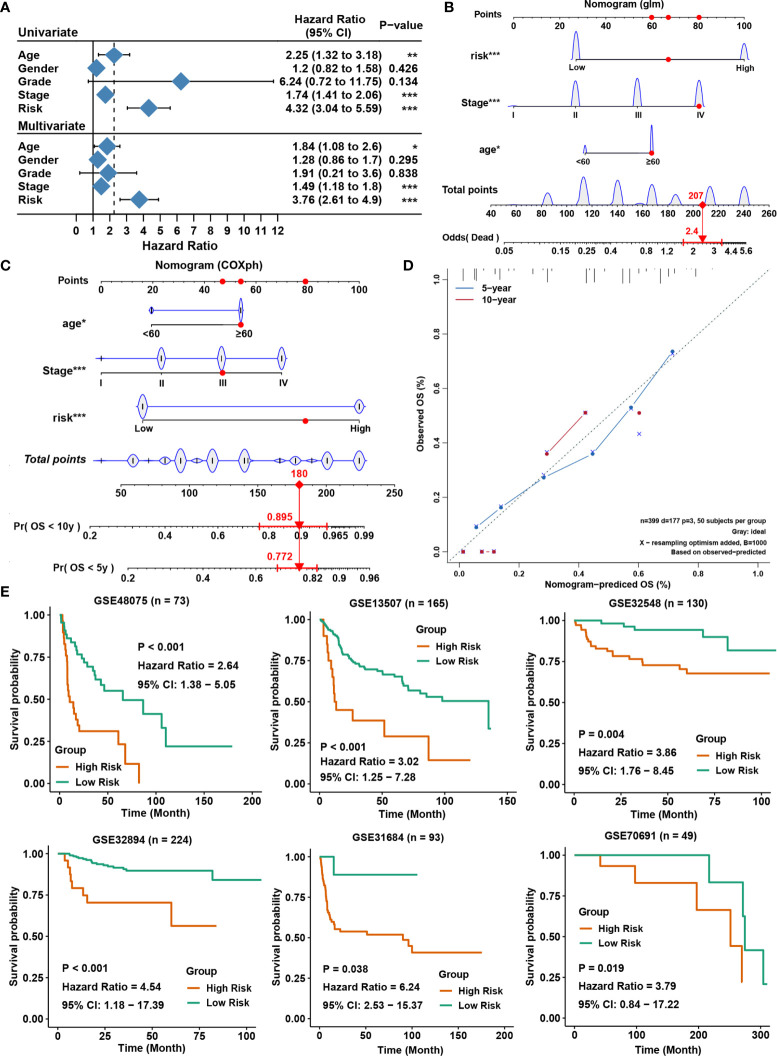
Construction of a predictive nomogram and external validation. **(A)** Univariate and multivariate Cox analyses of clinicopathological characteristics and gene signatures with overall survival in the TCGA BLCA cohort. **(B)** Nomogram to predict the odds of mortality of BLCA patients. **(C)** Nomogram to predict the 5‐y and 10‐y overall survival of BLCA patients. **(D)** Calibration curve for the overall survival nomogram model in the TCGA BLCA cohort. The dashed diagonal line represents the ideal nomogram, and the blue line and red line represent the 5‐y and 10‐y observed nomograms, respectively. **(E)** Validation of the writer-related gene signature in six external BLCA datasets for overall survival. *P < 0.05, **P < 0.01, ***P < 0.001.

We employed a nomogram to construct a method which were able to forecast the survival chance of a patient. Through the univariate and multivariate analyses between the included indicators and the OS (**Figure 4A**), we constructed a nomogram to forecast the odds of mortality of patients with generalized linear regression (**Figure 4B**) and to foresee the OS rates for five and ten years in TCGA using the Cox regression algorithm (**Figure 4C**). The predictors included the writer-related gene signature, age of patients and stage of patients. Compared to an ideal model in the entire cohort, the calibration plots for OS rates of five and ten years were predicted. (**Figure 4D**). Univariate and multivariate cox regression analysis of these ten genes was conducted in the six validation cohorts. We found most of them were prognosis related and the coincidence index of the multivariate model is mostly over 0.7 in these validation cohorts (**Figures S1, S2A, B**). Moreover, equivalent analyses were also performed on the six external validation groups GSE13507, GSE32548, GSE32894, GSE48075, GSE70691 and GSE31684 and we calculated the risk scores of every patient based on the signature. And patients in the higher‐risk group exhibited an importantly lower OS rate (**Figure 4E**). Time-dependent receiver operating characteristic curve were also conducted to judge the diagnostic ability of the model in these six validation cohorts. The result showed that the model also has a good diagnostic ability in the validation cohorts (**Figures S1–2C**). This was in accordance with conclusions deriving from the training set, showing the writer-related gene signature could accurately forecast the survival of patients with BLCA.

### Functional Characteristics of the Writer-Related Gene Signature

**Figure 5:**
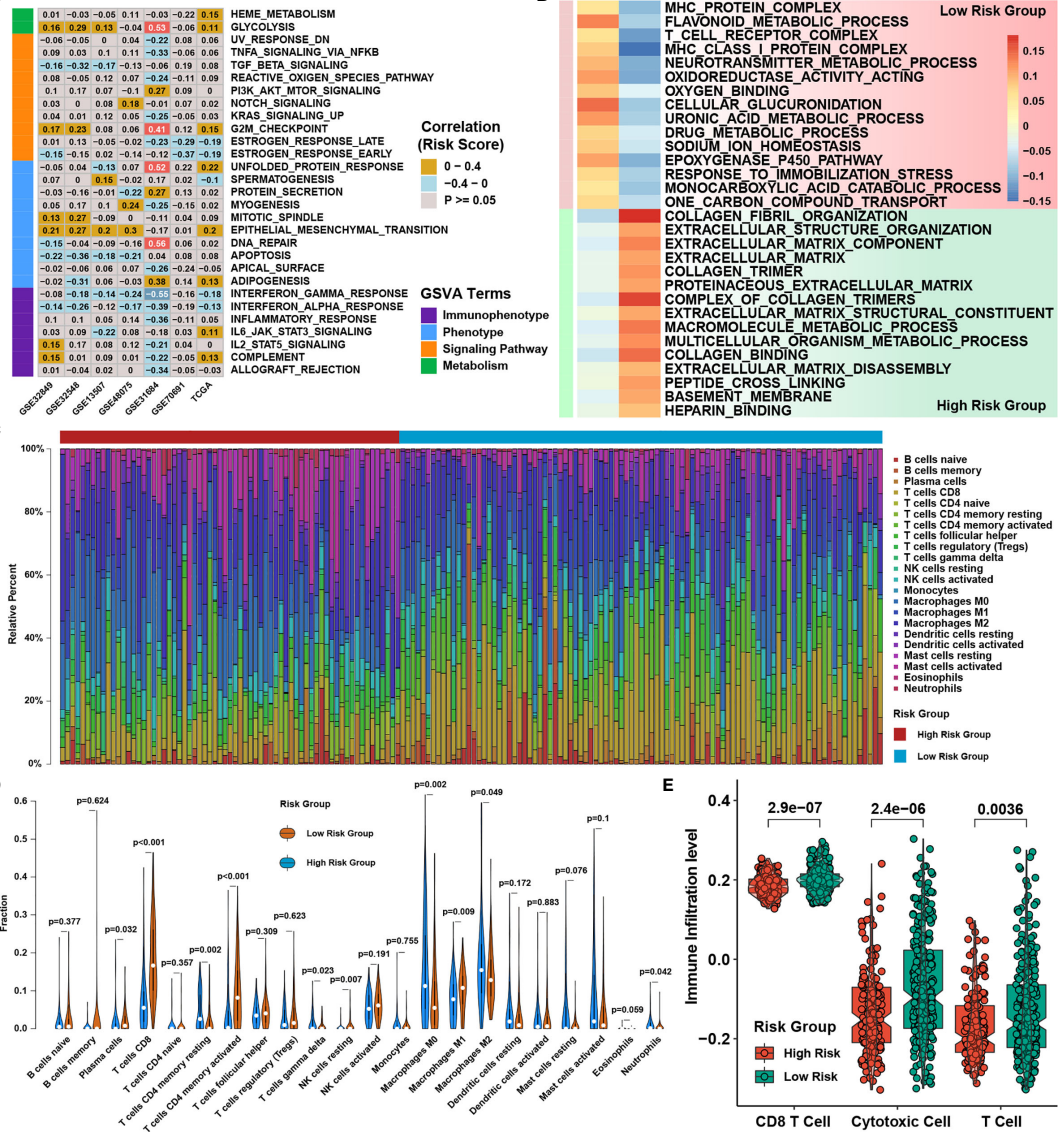
Functional characteristics of the writer-related gene signature. **(A)** Correlation analysis between the GSVA score for curated pathways and risk score in seven independent datasets. **(B)** GSEA revealed distinct enriched gene sets between risk score groups. In the heatmap, rows represent the 30 selected gene sets, and columns represent the consensus scores for each group. **(C)** The distribution of 22 immune cells calculated by CIBERSORT in the TCGA BLCA cohort. **(D)** Violin plot of the relative infiltration level of immune cells in the TCGA BLCA cohort. **(E)** The abundance of CD8 T cells, cytotoxic cells and T cells assessed by ssGSEA in different risk groups.

In order to find the possible mechanism of this gene signature, we performed GSVA on six validation cohorts and the TCGA cohort to assess the alteration of pathways (**Figure 5A**). We discovered the risk score was consistently positively associated with glycolysis and the EMT pathway, which was highly associated with tumor metastasis, and negatively correlated with apoptosis and the interferon-γ/α response pathways. For further validation of pathway alterations, GSEA was conducted on the TCGA cohort, and we found that T cell receptor complex- and MHC protein complex-related pathways were highly gathered in the group of low risk (**Figure 5B**). Subsequently, the immune infiltration in BLCA in the high‐risk and low‐risk groups was researched using of R package CIBERSORT. We used a bar plot to show the proportion of 22 immune cells in each subgroup (**Figure 5C**). The results demonstrated CD8 T cells, activated memory CD4 T cells, and M1 macrophages were highly infiltrated in the low-risk group and that M2 macrophages were highly enriched in the group of high risk (**Figure 5D**). The relative infiltration levels of T cells, cytotoxic cells and CD8 T cells were assessed using the ssGSEA approach, and the results showed that all of them were highly infiltrated in the low-risk group (**Figure 5E**). The results indicated the reason why the high-risk group had a poor prognosis might be associated with EMT-related tumor metastasis and the better prognosis of the low-risk group might be connected to higher CD8 T cell and other antitumor immune cell infiltration levels, which might be a response to immunotherapy.

### Potential Therapeutic Value of the Writer-Related Gene Signature

**Figure 6:**
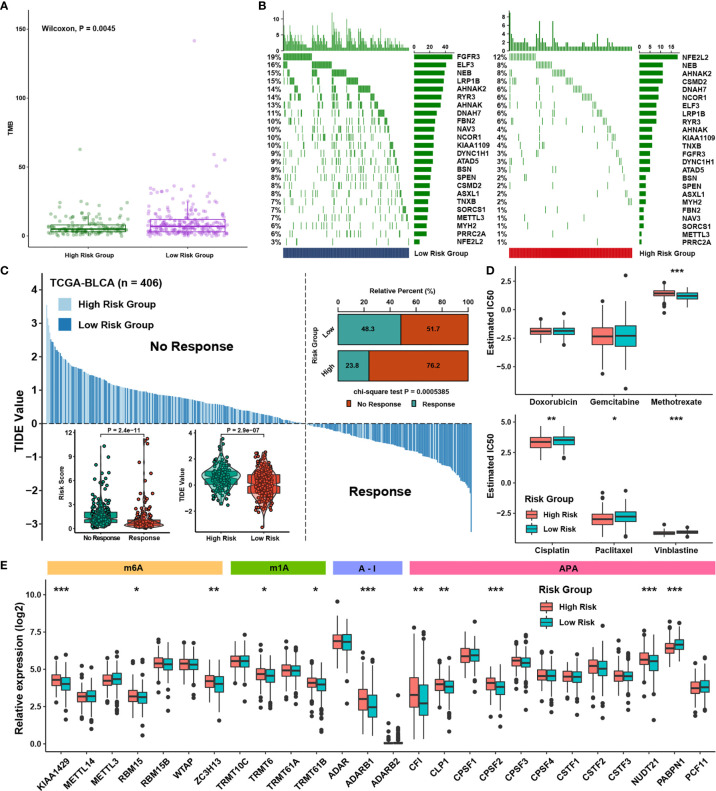
Potential therapeutic value of the writer-related gene signature. **(A)** The abundance of TMB in different risk groups. **(B)** Waterfall plot of tumor somatic mutations in patients with high risk scores and low risk scores. The columns represent individual patients. The upper bar plot shows TMB, and the number on the left indicates the mutation frequency in each gene. **(C)** The TIDE value of BLCA samples in TCGA is shown for different risk score groups, and the chi-square test used to assess significant differences is shown in the upper right. The risk scores and TIDE values in different response groups and risk groups are shown in the bottom left. **(D)** The estimated IC50 values of six commonly used chemotherapy drugs are shown in different risk groups. **(E)** The expression of 26 writer genes is shown in different risk groups. The asterisks represent the statistical P-value (*P < 0.05; **P < 0.01; ***P < 0.001).

It has been widely reported patients with a high tumor mutation burden (TMB) may benefit from immunotherapy due to more neoantigens (32). By analyzing the mutation annotation files of the TCGA BLCA cohort, we discovered the group of low risk owned a higher TMB than the group of high risk (**Figure 6A**), implying the low-risk group might benefit from immunotherapy. Then the distribution variation of somatic mutations between low- and high-risk scores in the cohort of TCGA-BLCA were analyzed utilizing the R package “maftools”. Just as the **Figure 6B** demonstrated, the high-risk-score group showed a less extensive tumor mutation burden than low-risk-score group. To predict the immune response of BLCA patients, patients were divided into response and no response groups with TIDE values, and a chi-square test revealed the low-risk-score group may have higher reactivity to immunotherapy (**Figure 6C**). We found that the risk score was lower in the response group and that the TIDE value was higher in the group of high risk. In order to explore the association between the risk scores and drug response, we evaluated the estimated IC50 value of six commonly used chemotherapy drugs, namely, cisplatin, gemcitabine, doxorubicin, methotrexate, paclitaxel and vinblastine, in the cohort of TCGA-BLCA (**Figure 6D**). We discovered the high-risk group may be more sensitive to treatment with cisplatin, paclitaxel and vinblastine, while the low-risk group might be more susceptible to treatment with methotrexate. Together, these results showed RNA modification writer genes were related to drug sensitivity. Finally, we compared the distributions of writer genes between the groups of high risk and low risk. And we found that differentially expressed writer genes had all four types of RNA modification patterns (**Figure 6E**). Thus, the writer-related risk score might be a potential biomarker for constructing both suitable and effective treatment strategies.

## Discussion

**Figure 7:**
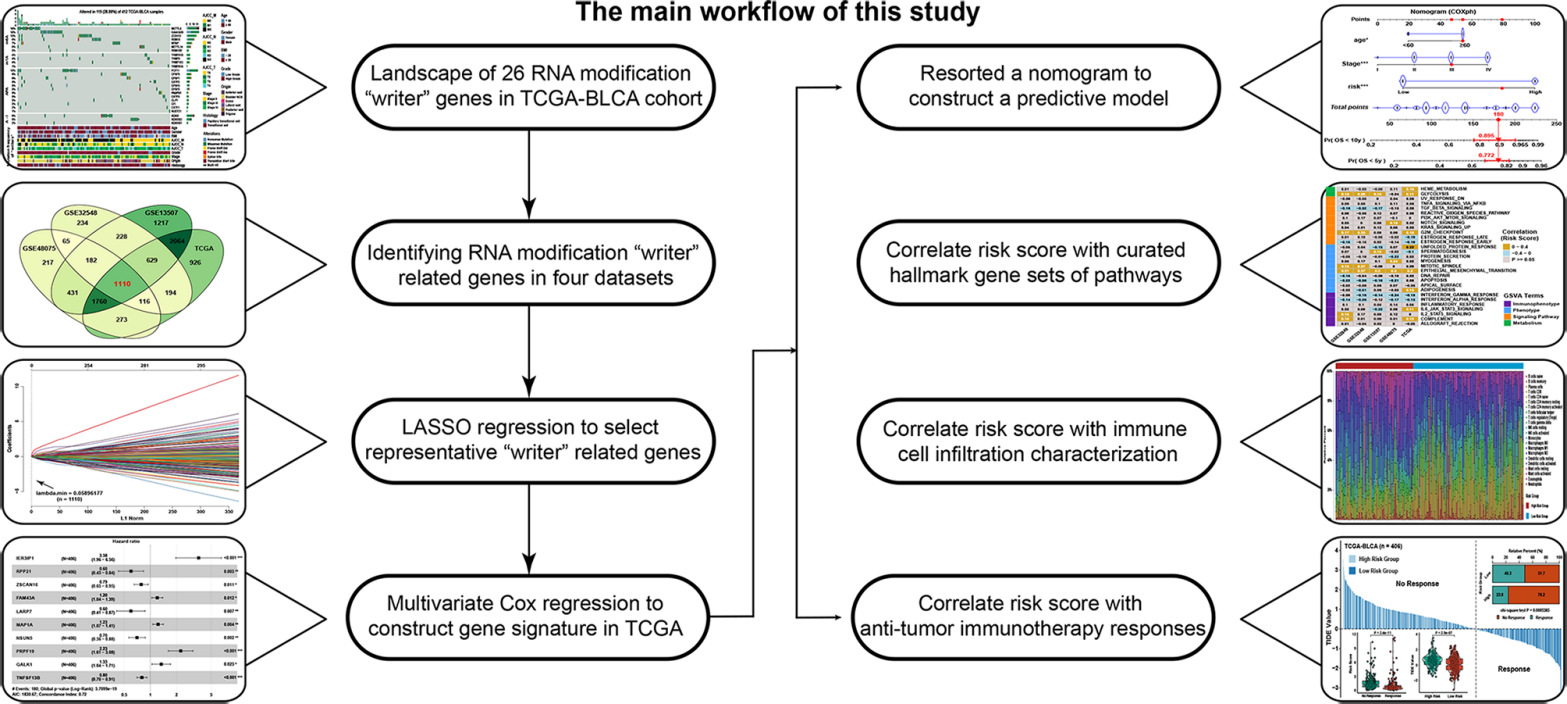
Flow chart of this study.

Rising evidence demonstrates RNA modifications have a significant function in antitumor activity, innate immunity and inflammation by interacting with distinct writers. Although many researches have concentrated in studying a single type of RNA modification writer gene, the mutual relationship and function of different types of writer genes in BLCA was not completely explored. This article analyzed four types of RNA modification patterns, namely, m6A, m1A, APA, A-I RNA editing and flowchart of this study was shown in **Figure 7**. First, we depicted the landscape of these four types of writer genes at the genetic and transcriptional levels and their correlations in BLCA. Then, we found writer-related genes in four independent datasets and constructed a scoring model, i.e., a writer-related gene signature, to predict the prognostic risk of individual patients. The high-risk-score group was associated with a worse prognosis and enriched in activated EMT-related pathways. The infiltration levels of immune cells in the tumor microenvironment was greatly increased in the low-risk-score group, which indicated a better immunotherapy benefit, consistent with the TIDE value and TMB score results.

EMT is related to metastasis of tumor and drug resistance (33), and M2 macrophages can suppress proliferation and differentiation of T cells, but advancing the proliferation of tumor cells and tumor metastasis (34). A former research demonstrated that M2 macrophages were related to EMT and enhanced both the invasion and migration of tumor cells in later stages of tumor (35, 36). These changes may increase macrophage M2 infiltration in the microenvironment of tumor, promoting the invasion and metastasis of BLCA cells. From our perspective, the activation of EMT-related pathways and high infiltration of M2 macrophages might be the reasons that the high-risk group was associated with poor survival. We suggested that cisplatin, paclitaxel and vinblastine might be more effective in patients with high risk scores. In contrast, the low-risk group had significantly longer survival and higher infiltration of CD8 T and cytotoxic cells and upregulated apoptosis and interferon response pathways. It has been reported that M1 macrophages can secrete INF-γ, IL-16, IL-12, and other proinflammatory cytokines, starting the inflammatory response and killing tumor cells (34). These traits were enriched in the group of low risk, showing patients in the low-risk group might benefit from immunotherapy.

The link between the risk score and writer genes also verified the important function of RNA modification writer genes. The expression of METTL3, an m6A writer gene, was higher in the high-risk group and tumor samples, which has been reported to regulate the cell cycle, cancer stem cells, and metabolism, resulting in tumor cell proliferation, metastasis, and tumorigenesis (37, 38). PABPN1, a suppressor of APA, might suppress tumor aggressiveness by releasing cancer cells from microRNA-mediated gene regulation (31) and was higher in the low-risk group. We found that many writer genes were differentially expressed between the low- and high-risk groups, which might provide novel epigenetic therapeutic targets in BLCA. For the ten candidate writer-related genes, there are few studies in bladder cancer, but some of their biological functions have been explored and verified in other studies. TNFSF13B has been reported to be a microenvironment-related gene that could predict poor prognosis in kidney renal clear cell carcinoma (39, 40). High expression of NSUN5 has been reported to promote cell proliferation *via* cell cycle regulation in colorectal cancer (41). LARP7 functions as a tumor suppressor gene in gastric cancer and can suppress P-TEFb activity to inhibit breast cancer progression and metastasis (42, 43). Although these writer-related genes were highly coexpressed with writer genes, the specific relationship should be further experimentally confirmed both *in vitro* and *in vivo*.

At last we demonstrated the latent therapeutic effects of the writer-related gene signature in BLCA, which was associated with immunotherapy. With higher tumor mutation burden and immune cell infiltration, the low-risk group owned a greater possibility of responding to immunotherapy. Regarding the mutated genes between risk groups, we found that FGFR3 mutations were more frequent in the low-risk group. It has been reported FGFR3 mutations were more common in noninvasive BLCA and related to a better BLCA prognosis, and patients with FGFR3 mutant tumors could benefit from anti-FGFR3 therapy (44, 45). This also reflected the low-risk group experienced a better prognosis result compared to the high-risk group, and a more therapeutic regimen could be selected for the patients of low-risk group. However, activated EMT-related pathways in the high-risk group caused this subgroup to have a higher propensity for metastasis and a lack of corresponding therapeutic targets. By identifying the estimated IC50 of antitumor drugs and enabling individualized immunotherapy, our research offers novel feasibility for advancing the effect of chemotherapy for patients with BLCA.

Although immunotherapy has become a novel strategy for oncological treatment, studies have found only approximately 20% of solid tumor patients could gain benefit from this kind of treatment, while others were not (46). Therefore, multiple studies have focused on identifying and verifying indicators that can accurately forecast efficacy of immunotherapy treatment. Some clinical parameters, including PD-L1 expression (47), CD8+ T cells (48), TMB (32), and microsatellite instability (MSI) (49), are used to predict immunotherapy treatment efficacy. It has also been reported that an immune-associated gene signature correlates with the immunophenotype, which could predict the anti-PD-L1 effect of urothelial cancer (50). While all of these factors or signatures were based on indicators associated with the immune response, our findings suggested that RNA modification patterns could also possess potential biological functions in predicting the efficacy of immunotherapy.

## Conclusions

In conclusion, our comprehensive research in RNA modification writers demonstrates a possible way how these writers influence the tumor microenvironment and their relation to the prognosis of patients with BLCA. We built a writer-related gene signature to document the crosstalk and functional roles of the writers both in transcription and posttranscriptional aspects and found their therapeutic effects in immunotherapy and target therapy. Moreover, this research also stresses the significant clinical implications of RNA modifications and will aid in the growth of personalized therapeutic strategies for BLCA patients.

## Data Availability Statement

Publicly available datasets were analyzed in this study. This data can be found here:

https://www.ncbi.nlm.nih.gov/geo/query/acc.cgi?acc=GSE13507

https://www.ncbi.nlm.nih.gov/geo/query/acc.cgi?acc=GSE32548

https://www.ncbi.nlm.nih.gov/geo/query/acc.cgi?acc=GSE32894

https://www.ncbi.nlm.nih.gov/geo/query/acc.cgi?acc=GSE48075

https://www.ncbi.nlm.nih.gov/geo/query/acc.cgi?acc=GSE70691

https://www.ncbi.nlm.nih.gov/geo/query/acc.cgi?acc=GSE31684

https://xenabrowser.net/datapages/?dataset=TCGA-BLCA.cnv.tsv&host=https%3A%2F%2Fgdc.xenahubs.net&removeHub=https%3A%2F%2Fxena.treehouse.gi.ucsc.edu%3A443https://xenabrowser.net/datapages/?dataset=TCGA-BLCA.htseq_fpkm.tsv&host=https%3A%2F%2Fgdc.xenahubs.net&removeHub=https%3A%2F%2Fxena.treehouse.gi.ucsc.edu%3A443.

## Author Contributions

PZ and ZL conducted the formal analysis and wrote the original draft. DW and YL participated in software. YJX conducted data curation. ZL conducted visualization analysis and software operating. PZ and ZL contributed to writing, reviewing, and editing the article. YJX and YFX revised the manuscript and provided funding acquisition. All authors contributed to the article and approved the submitted version.

## Funding

The study was supported by National Natural Science Foundation of China (Grants No. 000004608 and 900002627).

## Conflict of Interest

The authors declare that the research was conducted in the absence of any commercial or financial relationships that could be construed as a potential conflict of interest.

## Publisher’s Note

All claims expressed in this article are solely those of the authors and do not necessarily represent those of their affiliated organizations, or those of the publisher, the editors and the reviewers. Any product that may be evaluated in this article, or claim that may be made by its manufacturer, is not guaranteed or endorsed by the publisher.

## Abbreviations

BLCA, Bladder cancer; GEO, Gene-Expression Omnibus; TCGA, The Cancer Genome Atlas; TPM, Transcripts per kilobase per million; UTRs, Untranslated regions; CNV, Copy number variation; LASSO, Least absolute shrinkage and selection operator; ROC, Receiver operating characteristic; GSVA, Gene set variation analysis; KEGG, Kyoto Encyclopedia of Genes and Genomes; ssGSEA, Single-sample gene set enrichment analysis; TIDE, Tumor Immune Dysfunction and Exclusion; NSCLC, Non-small cell lung cancer; GDSC, Genomics of Drug Sensitivity in Cancer; IC50, Half-maximal inhibitory concentration; OS, Overall survival; MSI, microsatellite instability; TMB, Tumor mutation burden.
