# Supplementary material for: Scoring System Based on RNA Modification Writer-Related Genes to Predict Overall Survival and Therapeutic Response in Bladder Cancer
Source: Front Immunol. 2021 Aug 26;12:724541. doi: 10.3389/fimmu.2021.724541 (PMC8427805; doi:10.3389/fimmu.2021.724541)
Supplement: Supplementary file 1 [file DataSheet_1.docx]

**Title Page**

**Title:** Scoring system based on RNA modification writer-related genes to predict overall survival and therapeutic response in bladder cancer

Pu Zhang^1, #^, Zijian Liu^2, #^, Decai Wang^3^, Yunxue Li^1^, Yifei Xing^1*^, Yajun Xiao^1, *^

^1^ Department of Urology Surgery, Union Hospital, Tongji Medical College, Huazhong University of Science and Technology, 1277 Jiefang Avenue, Wuhan, 430022, China.

^2^ Department of Head and Neck Oncology and Department of Radiation Oncology, Cancer Center and State Key Laboratory of Biotherapy, West China Hospital, Sichuan University, Chengdu, Sichuan 610041, China.

^3^ Department of Emergency Surgery, Union Hospital, Tongji Medical College, Huazhong University of Science and Technology, 1277 Jiefang Avenue, Wuhan, 430022, China.

^#^ These two authors contributed equally to this work.

* Corresponding author: Yajun Xiao, M.D.

E-mail: [1984XH0663@hust.edu.cn](mailto:1984XH0663@hust.edu.cn) Phone: 15607123366

* Corresponding author: Yifei Xing

E-mail: Yifei_Xing@163.com Phone: 13296517036

**
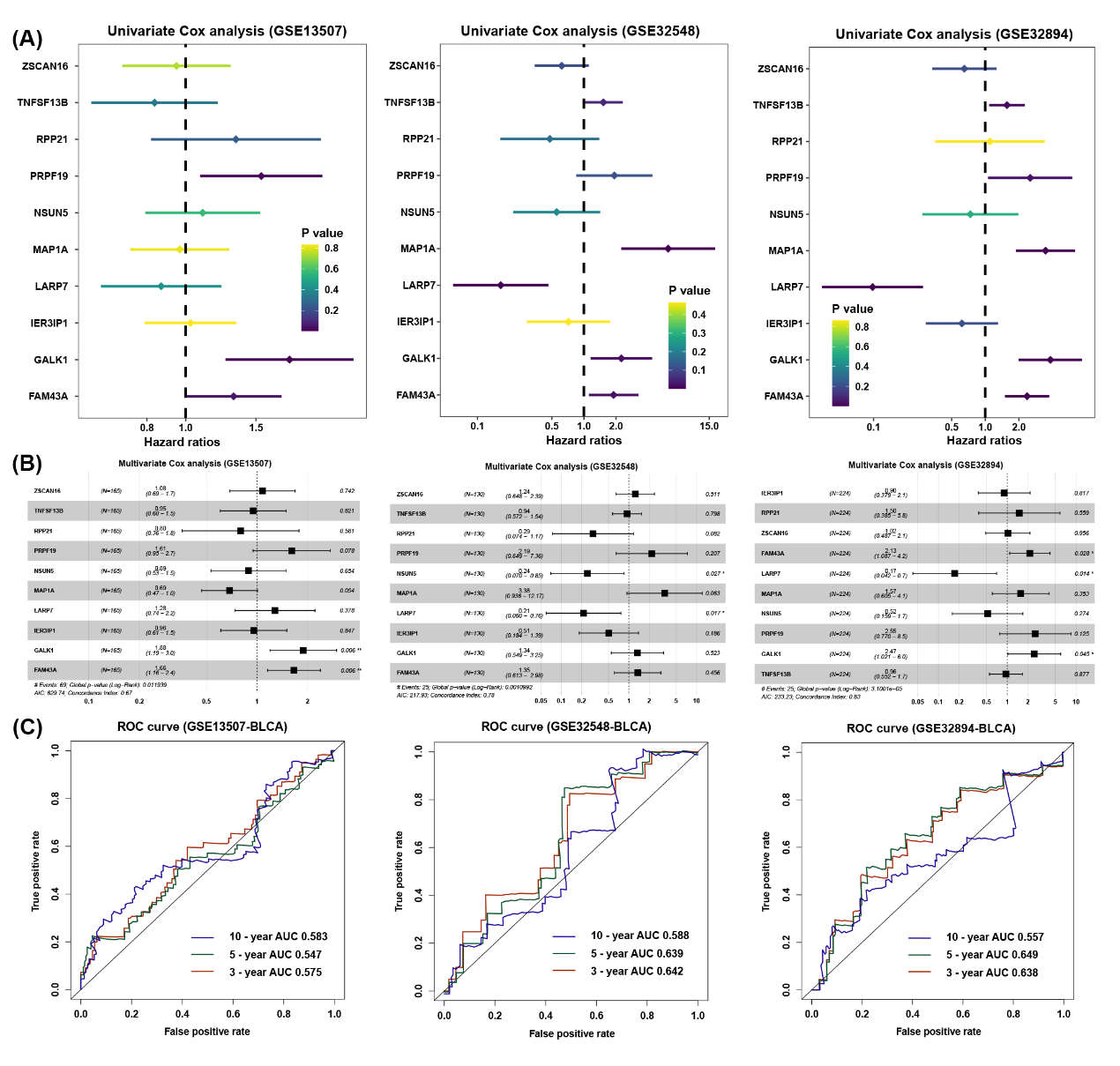
**

**Fig. S1 Validation of ten writer-related genes signature.** **a** Univariate Cox regression analysis of ten writer-related genes in validation cohorts. **b** Multivariate Cox regression of ten writer-related genes in validation cohorts. **c** ROC curves plotted for 3‐, 5‐ and 10‐y overall survival in validation cohorts.


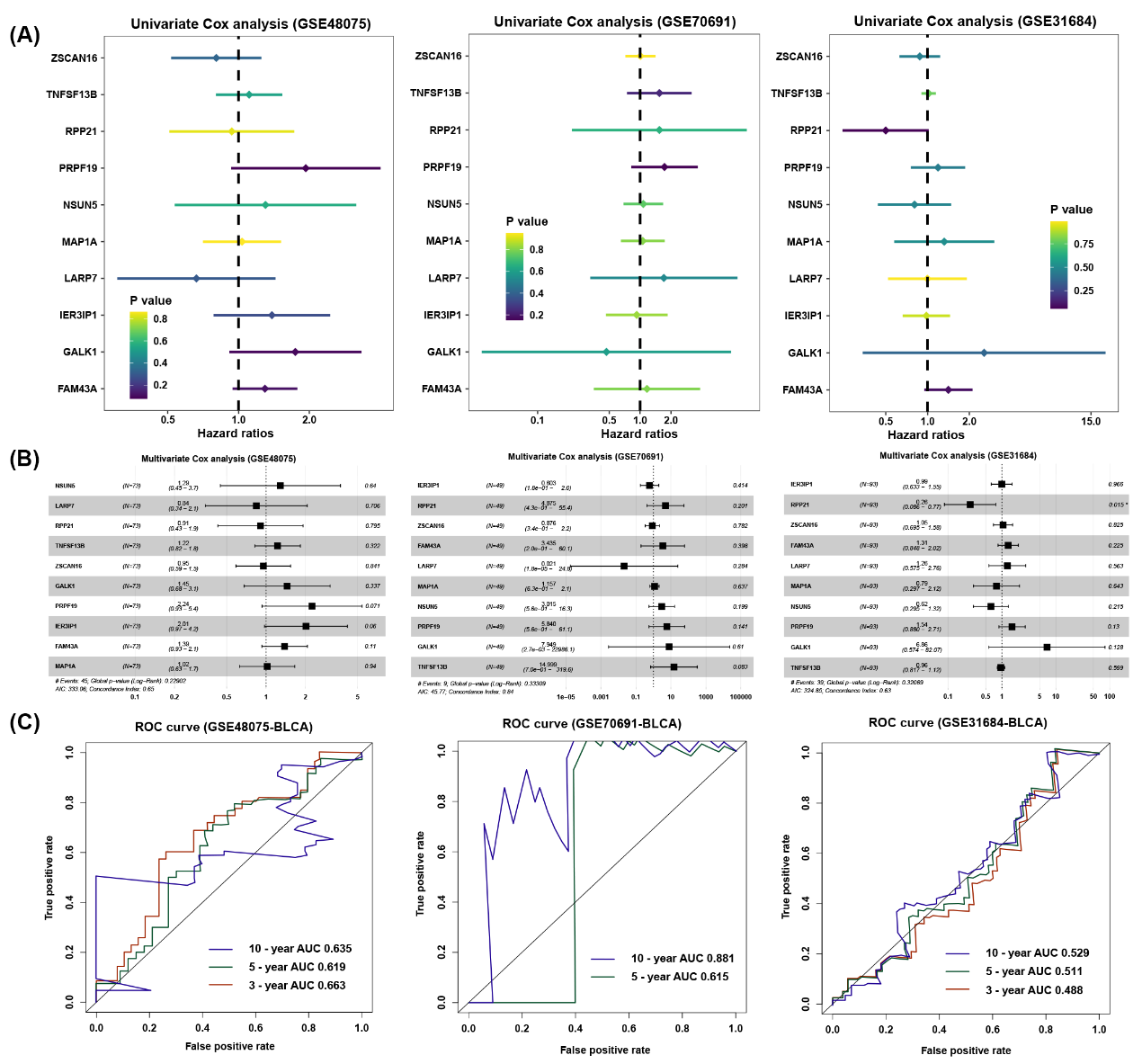


**Fig. S2 Validation of ten writer-related genes signature.** **a** Univariate Cox regression analysis of ten writer-related genes in validation cohorts. **b** Multivariate Cox regression of ten writer-related genes in validation cohorts. **c** ROC curves plotted for 3‐, 5‐ and 10‐y overall survival in validation cohorts.
